# Antimicrobial Resistance Genes in Respiratory Bacteria from Weaned Dairy Heifers

**DOI:** 10.3390/pathogens13040300

**Published:** 2024-04-03

**Authors:** Sarah Depenbrock, Cory Schlesener, Sharif Aly, Deniece Williams, Wagdy ElAshmawy, Gary McArthur, Kristin Clothier, John Wenz, Heather Fritz, Munashe Chigerwe, Bart Weimer

**Affiliations:** 1 Department of Veterinary Medicine and Epidemiology, School of Veterinary Medicine, University of California Davis, Davis, CA 95616, USA; 2 Department of Population Health and Reproduction, 100K Pathogen Genome Project, School of Veterinary Medicine, University of California Davis, Davis, CA 95616, USA; cschlesener@ucdavis.edu; 3 Veterinary Medicine Teaching and Research Center, School of Veterinary Medicine, University of California Davis, Tulare, CA 93274, USA; 4 Department of Internal Medicine and Infectious Diseases, Faculty of Veterinary Medicine, Cairo University, Giza 12613, Egypt; 5 Swinging Udders Veterinarian Services, Galt, CA 95632, USA; 6 California Animal Health and Food Safety Laboratory, School of Veterinary Medicine, University of California Davis, Davis, CA 95616, USA; 7 Field Disease Investigation Unit, Department of Veterinary Clinical Sciences, College of Veterinary Medicine, Washington State University, Pullman, WA 99163, USA

**Keywords:** *Mannheimia*, *Pasteurella*, *Histophilus*, bovine, airway, fluoroquinolone, ceftiofur, macrolide, tetracycline

## Abstract

Bovine respiratory disease (BRD) is the leading cause of mortality and antimicrobial drug (AMD) use in weaned dairy heifers. Limited information is available regarding antimicrobial resistance (AMR) in respiratory bacteria in this population. This study determined AMR gene presence in 326 respiratory isolates (*Pasteurella multocida*, *Mannheimia haemolytica*, and *Histophilus somni*) from weaned dairy heifers using whole genome sequencing. Concordance between AMR genotype and phenotype was determined. Twenty-six AMR genes for 8 broad classes of AMD were identified. The most prevalent, medically important AMD classes used in calf rearing, to which these genes predict AMR among study isolates were tetracycline (95%), aminoglycoside (94%), sulfonamide (94%), beta-lactam (77%), phenicol (50%), and macrolide (44%). The co-occurrence of AMR genes within an isolate was common; the largest cluster of gene co-occurrence encodes AMR to phenicol, macrolide, elfamycin, β-lactam (cephalosporin, penam cephamycin), aminoglycoside, tetracycline, and sulfonamide class AMD. Concordance between genotype and phenotype varied (Matthew’s Correlation Coefficient ranged from −0.57 to 1) by bacterial species, gene, and AMD tested, and was particularly poor for fluoroquinolones (no AMR genes detected) and ceftiofur (no phenotypic AMR classified while AMR genes present). These findings suggest a high genetic potential for AMR in weaned dairy heifers; preventing BRD and decreasing AMD reliance may be important in this population.

## 1. Introduction

Dairy heifer rearing is a significant part of California’s USD 10 billion dairy industry [1]. Bovine respiratory disease (BRD) is the most common cause of mortality and indication for antimicrobial drug (AMD) use in weaned dairy heifers [2]. Antimicrobial resistance is highly prevalent in respiratory bacteria from weaned dairy heifers in California [3]; however, the genomic basis for AMR in respiratory isolates specifically from weaned dairy heifers has not been described. Weaned dairy heifers represent a unique production group, compared to pre-weaned calves or adult cattle, in which to study BRD and AMR. This population has unique management conditions that may influence BRD and AMR; they have recently been weaned off milk feeding, which is stressful [4,5], and many have received prior treatment with AMD during the pre-weaned period for BRD or diarrhea [3,6,7,8,9,10], and have been moved to group housing where they may be comingled with calves from other farms, which represents stress and a risk for pathogen and AMR spread [3,11]. Antimicrobial resistance (AMR) in food animals is an animal health, welfare, and One Health concern [12,13,14,15].

Although the respiratory pasteurellaceae of cattle are not generally considered zoonotic pathogens in humans, AMR in respiratory bacteria of cattle represents many One Health risks. The use of AMD for the treatment of BRD affects other bacterial populations in the animal, including enteric zoonotic pathogens. The transfer of genetic elements that encode AMR between respiratory and enteric bacteria has been documented in cattle [16]. The CDC reports that more than 2.8 million AMR infections occur in the United States annually and that more than 35,000 people die as a result; multiple pathogens listed as AMR threats by the CDC are enteric pathogens, some of which are zoonotic such as Enterobacteriaceae and *Salmonella* spp. [17]. Studies around the globe have documented the transfer of AMR bacteria between cattle or other livestock species and many different human populations [18,19,20,21,22]. Transfer of AMR between livestock operations and the environment, via multiple different modes of transfer, has likewise been documented [23,24,25]. Additionally, the spread of AMR in respiratory pathogens between domestic and wild ruminants has likewise been documented [26].

The genomic basis of AMR is clinically important because it provides information about resistance mechanisms across groups of organisms, for many classes of AMD, in a single analysis. Whole genome sequencing (WGS) is one method by which AMR genes can be identified and can be used to determine total genetic diversity and molecular epidemiology and evaluate mechanistic insights into AMR. The advantage of whole genome sequencing compared to traditional in vitro susceptibility testing methods that determine AMR phenotype is that WGS provides data rapidly and examines all known AMR resistance genes in a single test. In contrast, culture and susceptibility testing methods are applied to a specific panel of AMD and assess a narrow range of pre-determined AMD dilutions. The increasing availability of WGS makes it a clinically useful and relevant methodology for sequence-based assessment of AMR. Workflows that provide reports for clinical consideration can be constructed [27,28,29], and this approach is becoming routine for AMR analyses in environmental samples [30]. The use of AMR gene presence to predict resistance phenotype has not been widely validated in multiple bacteria and within a genus [28]. Genotype predictions of phenotype are emerging with variable results; even within a single organism, the concordance of AMR genotype with phenotypic is variable [31,32,33]. Studies in Salmonella spp. and *Staphylococcus aureus* reported some agreement between AMR genotypic and phenotype [31,34,35]. Substantial discrepancies were identified between AMR phenotype and genotype in *Mannheimia haemolytica* respiratory isolates from beef cattle, depending on what drug was being analyzed [36]. Similarly, respiratory isolates *M. haemolytica*, *Pasteurella multocida*, *Histophilus somni*, and *Mycoplasma bovis* from pre-weaned dairy calves and feedlot cattle demonstrated discordance between genotype and phenotype based on the drug tested, bacterial species, and time point of sampling [37]. The variation among genetic AMR determinants in respiratory isolates between different aspects of the cattle industry, and differences in WGS within species of these organisms, suggest detailed information is needed to validate the use of WGS for predicting AMR phenotype in bacteria associated with BRD.

We hypothesized that AMR genes are highly prevalent and that AMR genotype and phenotype are well correlated in a study population of respiratory bacterial isolates from weaned dairy heifers in California. The objectives of this study were to (1) determine the AMR gene presence in respiratory bacterial isolates using WGS, and (2) determine the concordance between AMR genotype and AMR phenotype (using broth microdilution antimicrobial susceptibility testing) in the same isolates.

## 2. Materials and Methods

### 2.1. Study Design

A cross-sectional study was performed between June 2019 and February 2020 on a convenience sample of 6 dairy calf-rearing facilities across California’s Central Valley. A total of 360 heifers were sampled by deep nasopharyngeal swab (DNPS), as previously described [3,38]. Bacterial samples were submitted to the California Animal Health and Food Safety laboratory for culture and susceptibility testing of *P. multocida*, *M. haemolytica*, and *H. somni* isolates. The culture and susceptibility results were previously published in a study reporting AMR prevalence in the study population [3]. Banked isolates were then evaluated by WGS for this study and the results of WGS of *P. multocida*, *M. haemolytica*, and *H. somni* isolates were compared to the culture and susceptibility results.

### 2.2. Animals and Sample Sources

Original sample collection for this study population has previously been described [3]. Briefly, 360 heifers from 6 calf-rearing facilities were enrolled for sampling. In total, 3 of 6 calf-rearing facilities were multisource (ranging from 8 to 45 farm sources), 2 dairies raised their calves on site, and 1 dairy sent calves 1–7 d of age to a multisource calf-rearing facility (number of sources not reported) until weaning and weaned heifers returned to the source farm where they were sampled. This study was approved by the UC Davis Institutional Animal Care and Use Committee (protocol #20114); informed consent from herd management was obtained verbally prior to commencing study activities. Selection criteria included weaned dairy heifers in group pens (>3 months old) comingled for at least 2 weeks prior to sampling and less than 6 months old. Equal numbers of heifers with and without clinical signs of BRD based on a validated BRD Scoring system for weaned calves [39] were included. Samples were collected over two seasonal time points to include warmer (June–early October) and cooler (January–February) seasons. Respiratory bacterial isolates were collected by DNPS (double-guarded culture swabs, Reproduction Provisions LLC, Walworth, WI, USA), as previously described [38]. The DNPS were placed in Amies with charcoal transport media (CultureSwab Plus, BD BBL™, COPAN Italia 114 SpA, Brescia, Italy). Samples were stored in a cooler with wet ice during sampling and refrigerated at ~4 °C up to 48 h before submission. Selective culture and antimicrobial susceptibility testing for *P. multocida*, *M. haemolytica*, and *H. somni* was performed. A total of 361 isolates, consisting of 145, 119, and 97 *P. multocida*, *M. haemolytica*, and *H. somni* isolates, respectively, were collected. A loopful of pure growth was transferred from the MIC purity plate to the cryovial with treated beads and cryopreservative solution according to manufacturer instructions (Pro-Lab Diagnostics Microbank Cryovial Storage Systems) and stored at −70–80 °C (Panasonic VIP Plus) until WGS analysis.

### 2.3. Bacterial Culture and Susceptibility Testing

The methods for culture and susceptibility testing in this study population have previously been described [3], and are included in Supplementary Materials S1. Briefly, selective isolation was performed for *P. multocida*, *M. haemolytica*, and *H. somni*, and bacterial identification was confirmed by matrix-assisted laser desorption–ionization–time-of-flight (MALDI-TOF) mass spectrometry [40]. Antimicrobial susceptibility testing was performed using broth microdilution in BO-PO plates. Antimicrobial drugs selected for analysis in this study were based on the availability of the 2018 Clinical Laboratory Standard Institute (CLSI) breakpoints [41] that could be meaningfully interpreted with the AMD solution concentrations used for the respiratory isolates in the study and included tetracycline, tilmicosin, tildipirosin, gamithromycin, enrofloxacin, danofloxacin, florfenicol, spectinomycin, tulathromycin, penicillin, and ceftiofur. The AMD for which a MIC was obtained through broth microdilution but no applicable breakpoint was available included ampicillin, clindamycin, neomycin, sulfadimethoxine, tiamulin, trimethoprim–sulfamethoxazole, and tylosin; these AMD were analyzed separately. For the purposes of AMD class comparisons and MDR determination, when an isolate was classified as susceptible to all AMD tested in a class, the isolate was categorized as susceptible to that class; when an isolate was classified as resistant or intermediate to any drug in the class, the isolate was classified as resistant.

### 2.4. Whole Genome Sequencing (WGS)

A single bead from banked bacterial isolates was aseptically transferred to a 5% sheep blood agar plate (Hardy Diagnostics, Santa Maria, CA, USA). The bead was streaked across the primary quadrant and then removed. The plate was struck for single colony isolation with subsequent incubation aerobically at 37 °C (+2 °C) with 5% CO_2_ for 18–24 h. A loopful of pure growth (~10 µL) was transferred to Meuller–Hinton broth and incubated aerobically overnight at 37 °C for 18–24 h. Approximately 1 mL of suspension was transferred to a microcentrifuge tube in duplicate and the bacteria were pelleted by centrifugation at 16,000× *g* for 5 min. The supernatant was aspirated off and pellets were frozen at −80 °C until DNA was extracted, as described previously [42,43,44,45]. Briefly, the cell pellet was lysed with a hydrolytic enzyme cocktail for 30 min with DNA isolation completed using the Wizard Genomic DNA Purification kit (Promega, Madison, WI, USA), according to instructions for gram-negative bacteria. Duplicate bacterial pellets were combined with 600 µL Nuclei Lysis Solution and processed as a single sample. The final DNA pellet was rehydrated in 100 µL of 10 mM Tris-HCl pH 8.0. Genomic DNA quality was evaluated as described previously [46]. Briefly, purity for protein and organic contamination was conducted using a Nanodrop One UV-Vis Spectrophotometer (ThermoScientific, Waltham, MA, USA) using the A260/280 and the A260/230 of >1.5. Genomic DNA integrity was evaluated by genomic DNA TapeStation (Agilent 4200, Santa Clara, CA, USA) [46,47]. The DNA was stored at −20 °C until used for WGS.

The 361 bacterial isolates were prepared for WGS with the Illumina 2500 (San Diego, CA, USA), using the paired-end 150 method as previously described [27,42,48,49,50,51]. Briefly, high-quality gDNA was used to construct sequencing libraries with 400–550 bp inserts, followed by size selection to an average of 450 bp and sequenced to 50× depth per genome. Raw sequence information is available at the 100K Pathogen Genome Bioproject (PRJNA203445).

### 2.5. Informatic Tool Usage

Program packages and their dependencies were managed with Conda (Miniconda Software Distribution, Anaconda Inc., available at https://docs.conda.io/projects/miniconda, versions 4.8 to 23.10). The programs listed in this publication include the repository, version number, and package ID that are used on anaconda.org. Data organization was carried out with Python3 using Pandas data frames (version: conda-forge pandas=2.0.1=py311hab14417_1). Specific use of each tool is listed by functional need.

### 2.6. Genome Assembly and Quality Control

Genomic sequence data was processed with Trimmomatic {Ref. [52] Method1} (version: bioconda trimmomatic=0.39=1), using settings “trimmomatic PE {input} {output} ILLUMINACLIP:{adapters}:2:40:15 LEADING:2 TRAILING:2 SLIDINGWINDOW:4:15 MINLEN:50”, to remove low-quality sequence and sequencing adapters. Sequence data quality was reviewed with FastQC {Ref. [53] Method2} (version: bioconda fastqc=0.11.9=0). Genome assemblies were constructed with Shovill {Ref. [54]: Method3} (version: bioconda shovill=1.0.4=0) using the default options with the SPAdes assembler. Genome assembly quality was reviewed with CheckM {Ref. [55]: Method4} (version: bioconda checkm-genome=1.1.2=py_1), using the “lineage_wf” workflow. Each assembly’s depth of coverage was measured with Mosdepth {Ref. [56]: Method5} (version: bioconda mosdepth=0.3.1=h4dc83fb_1) using “fast-mode”. Quality control cutoffs for inclusion in the analyses were CheckM: >95% estimated completeness, <5% estimated contamination, within range 2–2.75 Mbases for assembly size, <300 contigs; Mosdepth: >20× mean coverage.

### 2.7. Investigating Contamination

Genomes were explored for contamination to identify species of the contaminants [57,58]. Trimmed sequence reads were assigned taxonomic identities with Kraken2 {Ref. [59]: Method6} (version: bioconda kraken2=2.0.8_beta=pl526hc9558a2_2), using standard settings (k-mer size = 35). Taxonomic assignment used standard Kraken2 database build of NCBI RefSeq genomes, following the Kraken2 manual protocol (https://github.com/DerrickWood/kraken2/blob/master/docs/MANUAL.markdown, accessed on 5 May 2021), incorporating the categories archaea, bacteria, viral, fungi, protozoa, and UniVec Core (built/downloaded 5 May 2021). Taxonomically assigned reads were statistically proportioned to the respective taxa at the species level with Bracken {Ref. [60]: Method7} (version: bioconda bracken=2.6.1=py38hed8969a_0). Braken species database was made from the Kraken2 database by standard protocol, using the parameters of k-mer size = 35 and read size = 150. Resulting assignments were organized into percentage abundance of sequence reads for each identified species per sample.

### 2.8. Identifying AMR Genes

Genome assemblies were scanned for AMR genes using the Resistance Gene Identifier (RGI) software (version: bioconda rgi=5.1.1=py_0) with the Comprehensive Antibiotic Resistance Database (CARD) (version: 3.1.1 released 29 January 2021) {Ref. [61]: Method8}. From CARD, identified genes’ information regarding types/classes of drug resistance conferred and mechanism of action were obtained.

### 2.9. AMR Gene Distribution Graphs

The prevalence of AMR genes was plotted, by species, in a heat map generated by Python script using the heat map function of the seaborn package (version: conda-forge seaborn=0.12.2=hd8ed1ab_0), and category labels were created with the “UpSet” function of the upsetplot package (version: conda-forge upsetplot=0.8.0=pyhd8ed1ab_0). Gene distribution by species was also plotted as a Venn diagram using the “venn3” function of the matplotlib Venn package (version: conda-forge matplotlib-venn=0.11.9=pyhd8ed1ab_0). Gene pairwise co-occurrence in genomes was presented as a proportion of samples with both genes relative to total samples with either or both genes (a Jaccard index). The pairwise gene co-occurrence matrix was clustered by the “squareform” and “linkage” function (method=‘single’) of the scipy.spatial.distance and scipy.cluster.hierarchy packages, respectively (version: conda-forge scipy=1.10.1=py311h939689b_1) and graphed with the clustermap function of the seaborn package.

### 2.10. Isolate Phenotype and AMR Genotype Classification

Standardized drug class categories were set for comparisons (Table S1). The class of beta-lactams was split into the sub-classes cephalosporin and penicillin for some comparisons where differentiation is relevant for clinical interpretation. The class macrolide+ combines the different chemical drug classes macrolide, lincosamide, and streptogramin as they are affected by many of the same resistance mechanisms. The AMR phenotype was determined by classification of MIC values as resistant or sensitive using the 2018 version of the CLSI breakpoints that were clinically applicable to cattle respiratory isolates for the respective drugs [3]. Briefly, isolates were classified as having a phenotype of ‘sensitive’ if their MIC value was within the CLSI range determined as susceptible, and classified as having a phenotype of resistant if within the CLSI range for resistant or intermediate. For drugs without a clinically applicable breakpoint, a similar binary phenotype interpretation was made as ‘low’ or ‘high’. For drugs tested over a range of concentrations, MIC value distributions were assessed to find distribution breakpoints of ‘low’ and ‘high’ by Jenks natural breaks classification [62]. For AMD that were only tested at one concentration, the phenotype ‘low’ was assigned if the isolate did not grow at the test drug concentration, or ‘high’ if it grew.

Isolates were classified as genotype resistant to each class of AMD for which AMR genes were identified by CARD in the isolate, and sensitive when no AMR genes were identified.

### 2.11. Statistical Analysis

Concordance between AMR phenotype and genotype was assessed. When comparing genotype predictive to phenotype, results were generated in the following categories: false positive [genotype(+) & phenotype(−)]; true positive [genotype(+) & phenotype(+)]; true negative [genotype(−) & phenotype(−)]; and false negative [genotype(−) & phenotype(+)]. The ability of AMR genes to predict AMR phenotypes was assessed from the confusion matrix style framework using sensitivity and specificity.

Comparisons were made individually for each drug tested against each gene present for the respective drug class. Comparisons were also made collectively by drug class, comparing resistance to any drug in the class to the presence of any gene that confers AMR to that class. Comparisons were made for bacterial species as well as all bacteria combined.

Statistical comparisons with a Python script used Panda’s data frames to sort and organize respective comparisons. Matthew’s correlation coefficient (MCC) was used to estimate the correlation between AMR genotype and phenotype. The MCC values range from −1 to 1, where positive correlation estimates represent increasingly agreeing genotype and phenotype AMR status classification, with +1 being perfectly in agreement, negative correlation estimates represent increasingly opposing genotype and phenotype AMR status classification, with −1 being perfectly opposing, while 0 implies perfect random genotype and phenotype pair classification. To test whether a specific MCC was statistically significant, McNemar’s test was used to account for the dependency between genotype and phenotype status observed from the same isolate. A McNemar’s exact test was used if the discordant cell counts (sum of false positives and false negatives) of a specific genotype–phenotype combination were sparse (n < 24). A 5% level of significance was observed to estimate significant correlation coefficients. Analyses were conducted using the SciPy package (version: conda-forge scipy=1.10.1=py311h939689b_1) to determine the significance of the associations between susceptibility testing and AMR gene ID from WGS. Additional statistical values were calculated by Python script with the standard formulas. McNemar’s standard and exact tests were conducted using the “contingency_tables.mcnemar()” function of the Statsmodels package (version: conda-forge statsmodels=0.14.1=py311hc9a392d_0). Additional statistical values were calculated by Python script with the standard formulas as listed in the column headings of Table S2.

Comparison of multidrug resistance between phenotype and genotype was carried out at a broad scale; the comparison was restricted to the 6 AMD classes that contain drugs tested with clinically relevant MIC breakpoints and were assessable from RGI/CARD genome scans. The set of classes assessed includes aminoglycosides, beta-lactams, fluoroquinolones, macrolides, phenicols, and tetracyclines. The cumulative count of drug-resistant classes by AMR genes increased if a gene was present for a respective class, and likewise for the cumulative count by MIC phenotype, increasing if an isolate was determined resistant for any drug in the respective class. Graphical comparisons were produced using the “histplot” function of the Seaborn package.

## 3. Results

A total of 326 genomes and corresponding assemblies passed quality control metrics and were used for analysis, including 130 *P. multocida*, 106 *M. haemolytica*, and 90 *H. somni*. Thirty-five samples were removed for quality control including some isolates where the bacterial identification (ID) provided by MALDI-TOF was in disagreement with the bacterial IDs determined using WGS; in some cases, multiple organisms were identified in a single sample (Tables S3 and S4).

**Figure 1:**
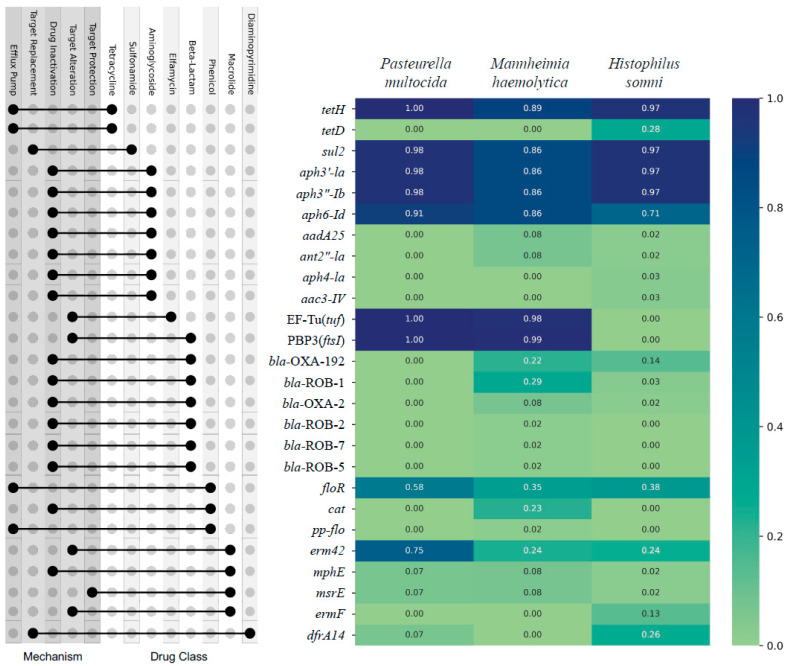
Prevalence of AMR genes identified by WGS of 326 respiratory isolates of *P. multocida*, *M. haemolytica*, and *H. somni* from weaned dairy heifers. The AMR gene is on the Y axis, and the prevalence of each gene is listed by bacterial species on the X axis; heat map with Likert scale demonstrates relative prevalence of each gene where darker blue corresponds to greater prevalence. The corresponding dot plot demonstrates which classes of AMD each gene is predicted to confer AMR (right dot) and the line links to the associated mechanism by which AMR is conferred (left dot). N = 130, 106, 90 *P. multocida*, *M. haemoltyica*, *H. somni* isolates, respectively.

There were 26 AMR genes, encoding resistance to 8 broad classes of AMD, identified from 326 isolates (Figure 1). The top nine most prevalent individual AMR genes across all three bacterial species, the percent of samples containing the gene, and the AMD class to which they predict AMR conference were as follows: tetH (95%, tetracycline), sul2 (94%, sulfonamide), aph3′-Ia (94%, aminoglycoside), aph3”-Ib (94%, aminoglycoside), aph6-Id (84%, aminoglycoside), PBP3(ftsI) (72%, beta-lactam), EF-Tu(tuf (72%, elfamycin), floR (45%, phenicol), and erm42 (44%, macrolide/lincosamide/streptogramin). When the identified AMR genes are sorted by class of AMD to which they predict conference of AMR, the most prevalent classes to which AMR would be conferred were as follows: tetracycline (95%), aminoglycoside (94%), sulfonamide (94%), beta-lactam (77%), elfamycin (72%), phenicol (50%), macrolide/lincosamide/streptogramin (44%), and diaminopyrimidine (10%) (Figure 1). 

**Figure 2:**
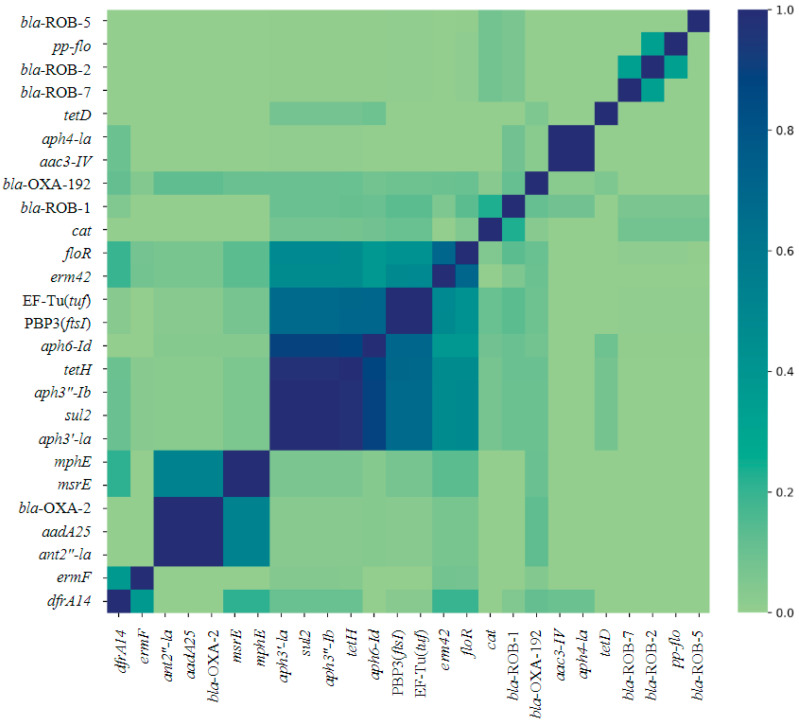
Jaccard similarity index heat map of co-occurrence of 26 AMR genes identified across respiratory bacterial isolates *P. multocida*, *M. haemoltyica*, and *H. somni* from weaned dairy heifers. Darker blue on the Likert scale indicates more frequent co-occurrence. N = 130, 106, 90 *P. multocida*, *M. haemoltyica*, *H. somni isolates*, respectively.

**Figure 3:**
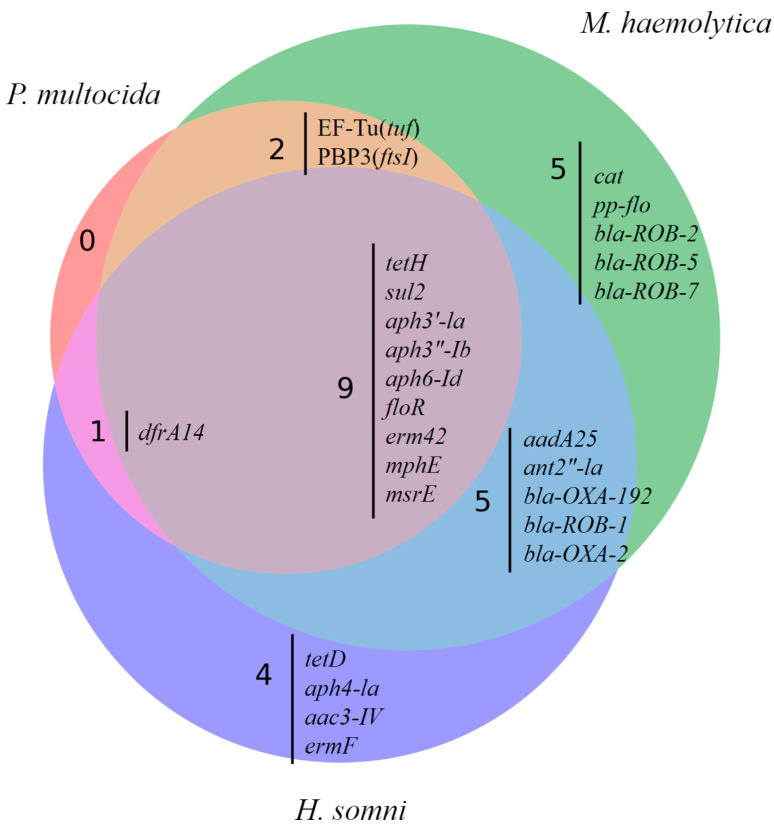
Venn diagram demonstrating co-occurrence of 26 AMR genes identified in respiratory bacterial isolates from weaned dairy heifers separated by bacterial species (*P. multocida*, *M. haemolytica*, *H. somni*). Each species of respiratory isolate is represented by a different circle. Co-occurrence of AMR genes between different species of bacterial respiratory isolate is represented where circles overlap. N = 130, 106, 90 *P. multocida*, *M. haemolytica*, *H. somni* isolates, respectively.

Multiple AMR genes co-occurred within isolates from each genus (Figure 2 and Figure 3). The AMR genes tetH, aph3”-Ib, aph3′-Ia, and sul2 occur together in most isolates (Figure 2). The largest cluster of gene co-occurrence included floR, erm42, EF-Tu(tuf), PBP3(ftsI), aph6-Id, aph3”-Ib, aph3”-Ia, tetH, and sul2 (Figure 2). These genes predict AMR to phenicol, macrolide, elfamycin, β-lactam (cephalosporin, penam cephamycin), aminoglycoside (all three aph genes listed), tetracycline, and sulfonamide class AMD, respectively.

**Figure 4:**
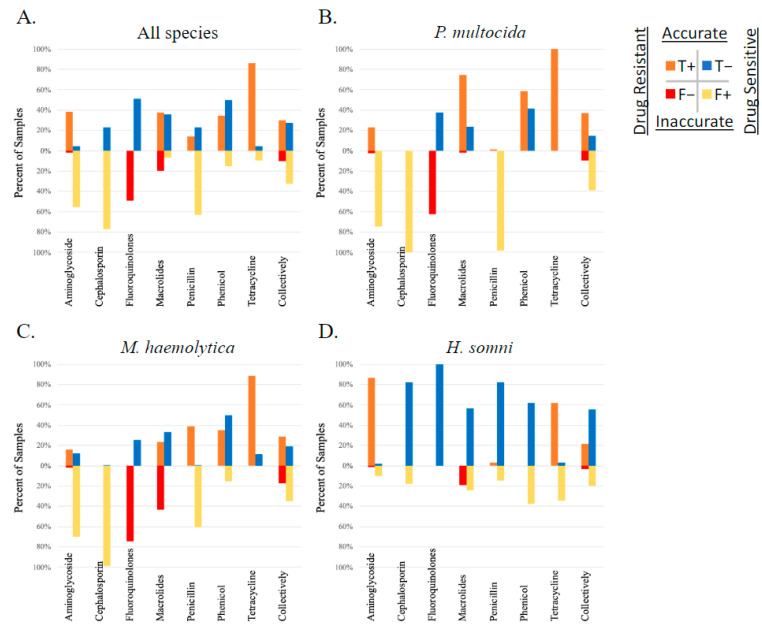
Performance of genotype to predict AMR phenotype. Presence of AMR genes determined by WGS compared to phenotype testing using susceptibility testing and breakpoint interpretation, in respiratory bacterial isolates *(P. multocida*, *M. haemolytica*, and *H. somni*) from weaned dairy heifers.

The correlation between AMR phenotype, as measured by susceptibility testing, and AMR gene ID was determined using Matthew’s correlation coefficient (MCC). The overall correlation between AMR gene ID and AMR phenotype of study isolates was low (median MCC = 0.15) when measured across all 326 isolates, between all AMR genes identified and the 11 AMD drugs analyzed for phenotype using susceptibility testing. The MCC ranged from −0.57 to 1 depending on the drug–gene–bacterial species combination analyzed. The likelihood that these correlations occurred by chance was tested using McNemar’s test. When analyzed by each isolate (*P. multocida*, *M. haemolytica*, or *H. somni*) and each AMR gene independently, the MCC was below 0.7 (the cutoff for excellent correlation) for most drug–gene–bacterial species combinations analyzed. The MCC was greater than 0.7 with a McNemar *p*-value < 0.5 for the following limited relationships representing lack of susceptibility (AMR phenotype): the bla-ROB-1 gene and penicillin AMR phenotype across all three species, and in *M. haemolytica* individually (MCC = 0.81, 0.84, respectively), the erm42 gene and tulathromycin AMR phenotype in *M. haemolytica* (MCC = 0.83), and the floR gene and florfenicol AMR measured across all three species (MCC = 0.80). It is important to note that when there are no discordant pairs (NDP), the MCC is perfect at 1 or −1; however, the 0 values in the McNemar equation created by NDP result in a P value of 1, and thus the significance of the correlation is inestimable. There were five comparisons where the MCC = 1, including between the floR gene and florfenicol AMR in both *P. multocida* and *M. haemolytica*, the erm42 gene and tildipirosin AMR in *P. multocida*, the bla-ROB-1 gene and penicillin AMR in *H. somni*, and the tetH gene and tetracycline AMR in *M. haemoltyica*. No MCC values were calculated for ceftiofur due to a lack of isolates classified as resistant or intermediate by MIC interpretation. The performance of AMR gene ID for the determination of respiratory bacterial isolate resistance phenotype is displayed in Figure 4.

The strength of the association between genotype and phenotype varied widely depending on the bacterial species, the AMD being tested, and the specific AMR gene. For example, identification of the erm42 gene was well correlated (MCC of 0.96, 0.92, and 1, respectively), highly sensitive (sensitivity of 0.98, 1, and 1, respectively), and specific (specificity of 1 for all) for prediction of the AMR phenotype in *P. multocida* to tilmicosin, gamithromycin, and tildipirosin; however, it was less well correlated (MCC of 0.26) and less sensitive (sensitivity of 0.31) for predicting phenotypic resistance to tulathromycin. Inversely, identification of the same erm42 gene in *M. haemolytica* was less well correlated (MCC of 0.4, 0.7, and 0.5, respectively) and less sensitive (sensitivity of 0.36, 0.61, and 0.42, respectively) for predicting phenotypic AMR for tilmicosin, gamithromycin, and tildipirosin and better correlated (MCC of 0.83) with greater sensitivity (sensitivity of 0.76) for the prediction of phenotypic AMR to tulathromycin. Table 1 displays the variation observed between AMR determined by gene ID and susceptibility testing within the macrolide sub-class, across the three bacterial species examined in this study. The MCC values, sensitivity, specificity, and McNemar’s test results comparing genotype to phenotype for all gene–AMD–bacterial species combinations are listed in Table S2.

**Figure 5:**
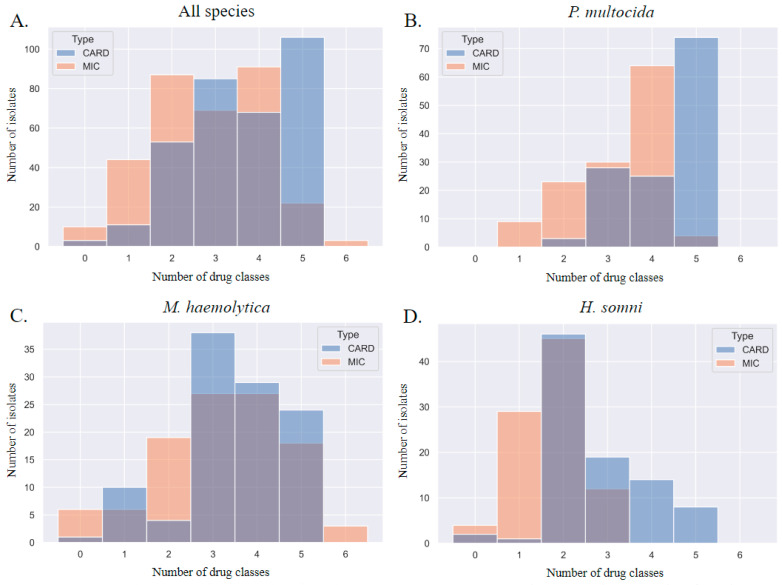
Step diagrams demonstrating the number of AMD classes to which an isolate was considered resistant based on MIC breakpoint interpretation or AMR gene ID. AMR determined by phenotype is peach, and genotype is blue. The AMD tested were grouped into classes based on AMR gene drug class-predicted resistance as follows: β-lactam (penicillin, ceftiofur), tetracycline (tetracycline), phenicol (florfenicol), macrolide (tulathromycin, tildipirosin, tilmicosin, gamithromycin), aminoglycoside (spectinomycin), fluoroquinolone (danofloxacin, enrofloxacin). N = 6 AMD classes. N = 326 isolates. Diagrams represent all three isolates combined (*P. multocida*, *M. haemolytica*, and *H. somni*) in panel (**A**), and separated by species for panels (**B**–**D**). CARD = AMR gene ID. MIC = AMR determined by MIC breakpoint analysis.

When each method was used to describe MDR, the MDR genotype consistently overestimated the MDR phenotype by one class. This discrepancy is visualized in Figure 5; the blue gene ID columns of the histograms are shifted to the right. The most prevalent patterns of AMR in *P. multocida* isolates were phenotypic resistance to four AMD classes (prevalence of 0.49) and genotypic AMR to five classes (0.57); the most prevalent patterns of AMR in *M. haemolytica* isolates were phenotypic and genotypic resistance to three AMD classes (0.25 and 0.36, respectively); the most prevalent patterns of AMR in *H. somni* isolates were phenotypic and genotypic resistance to two AMD classes (0.5 and 0.51, respectively).

**Figure 6:**
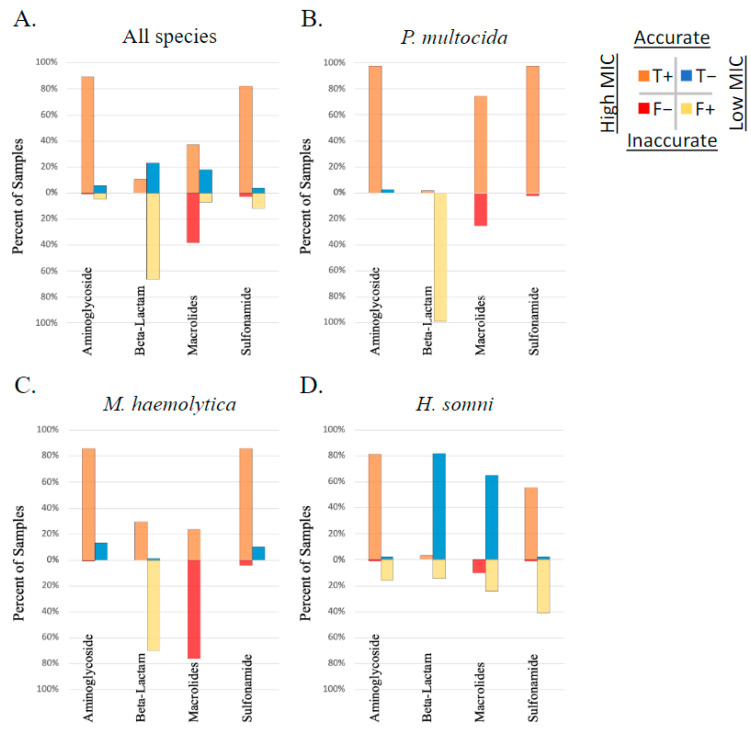
Performance of genotype to predict AMR phenotype of isolates with no applicable CSLI MIC breakpoint. Presence of AMR genes determined by WGS compared to phenotype testing using susceptibility testing and Jenks natural breakpoint classification for ‘high’ vs. ‘low’ MIC, in respiratory bacterial isolates (*P. multocida*, *M. haemolytica*, and *H. somni*) from weaned dairy heifers.

Figure 6 depicts the performance of AMR gene ID in predicting AMR (high vs. low MIC) against drugs with no applicable interpretation breakpoint (ampicillin, clindamycin, neomycin, sulfadimethoxine, tiamulin, trimethoprim–sulfamethoxazole, and tylosin). Combinations with high correlation included the following: the bla-ROB-1 gene was well correlated with a high MIC to ampicillin in *M. haemolytica* (MCC = 1); aph3′-Ia and aph3”-Ib were well correlated with high MIC to neomycin in *P. multocida* (MCC = 1); and the aph3′-Ia, aph3”-Ib, aph6-Id were well correlated with a high MIC to neomycin in M. haemolytica (MCC = 0.96). All other correlations between genotype and phenotype among AMD without an applicable breakpoint were moderate to low (MCC < 0.7).

## 4. Discussion

As hypothesized, AMR genes are highly prevalent in the study population. However, AMR genotype and phenotype are only well correlated for a subset of specific gene–bacteria–drug combinations.

The WGS of 326 bacterial respiratory isolates from weaned dairy heifers identified 26 known AMR genes associated with resistance to 8 broad classes of AMD. Genes predicted to confer resistance to medically important AMD commonly used in livestock including tetracyclines, sulfonamides, β-lactams, phenicols, and macrolides were among the most prevalent. Genes predicted to confer resistance to aminoglycosides were likewise frequently identified. There is a voluntary ban on the extra-label use of aminoglycosides in cattle [63]; however, several products containing neomycin sulfate are currently approved for use in cattle. Genes predicted to confer resistance to diaminopyridine class AMD (example: trimethoprim) were also commonly identified; there are no drugs from this class labeled for use in cattle, however, these AMD are used in an extra-label manner in some calf-rearing facilities, other veterinary species, and human medicine. Genes predicted to confer resistance to elfamycin class AMD were likewise frequently identified; this class of AMD has no drugs labeled for use in veterinary medicine; however, some AMD in this class are used in research. Genes predicted to confer AMR to tetracycline, aminoglycoside, phenicol, β-lactam, and macrolide class AMD were highly prevalent (>50%, up to 100% prevalence) in *P. multocida* isolates, similarly high in *M. haemolytica* isolates, and less prevalent in *H. somni* isolates. These Pasteurellaceae are clinically important respiratory bacteria associated with BRD in cattle, and *P. multocida* is particularly clinically important in calves with pneumonia. The prevalence of AMR genes in our study population raises concerns about the use of the medically important classes of drugs in this population of calves. Previous investigations have linked intensive rearing and AMD use in calves with AMR [64]. Although the analysis of AMD treatment history with AMR gene identification was beyond the scope of this study, it is reasonable to hypothesize that AMD use in pre-weaned dairy calves creates selective pressure on the respiratory Pasteurellaceae that may persist into the post-weaning period. This is problematic because respiratory disease is the primary cause of disease among weaned dairy heifers in the US [2]; the high prevalence of AMR genes in weaned dairy heifers reported in our study suggests that many of the drugs used for the treatment of BRD in this production group may continue to create selective pressure for AMR, and treatment may not be effective when these AMR genes are expressed. Additionally, there is a high rate of co-occurrence between AMR genes, which are commonly located in mobile genetic elements in Pasteurellaceae [65]; this could complicate efforts to decrease AMR because selective pressure on one class of AMD may also create selective pressure for other classes of AMD.

Genotype was variably correlated to phenotype and depended on which bacterial species and what drugs were compared. Concordance between genotype and phenotype was generally high for most aminoglycosides, macrolides, phenicols, and tetracycline.

Conversely, concordance between genotype and phenotype was poor for fluoroquinolones; no fluoroquinolone AMR genes were identified in the sample population, yet the AMR phenotype was observed. This finding is consistent with another study of California cattle respiratory isolates in which investigators reported that 20 of 64 isolates demonstrated resistance to a fluoroquinolone during susceptibility testing; however, no fluoroquinolone AMR genes were identified [37]. This incongruence may be due to a lack of representative single nucleotide polymorphism variants for these organisms in the database, known genes that are not currently classified as conferring AMR to fluoroquinolones, previously unrecognized genetic determinants of AMR acting in the study population, or phenotypic resistance associated with metabolic processes and not necessarily acquired genetic elements. It is also possible that other databases not explored in this study contain additional gene references; however, it was beyond the scope of our study to explore multiple AMR gene databases or gene-finding methodologies or to individually validate gene identities of patrial AMR gene matches.

Concordance was also poor between genotype and phenotype for cephalosporins. Six AMR genes identified predicted resistance to β-lactam AMD including the subclass cephalosporins, according to CARD database metadata (PBP3(ftsI), bla-ROB1, bla-ROB2, bla-ROB5, bla-ROB7 and bla-OXA-2); however, no isolates demonstrated a resistance phenotype to ceftiofur, a third generation cephalosporin. Although some bla-OXA genes confer limited resistance to penam type β-lactam AMD such as oxacillin, bla-OXA-2 is among those bla-OXA genes listed as extended-spectrum including the conference of resistance to cephalosporin class AMD. Some of these genes, such as PBP3(ftsI), were highly prevalent in *P. multocida* and *M. haemolytica* isolates (100 and 99%, respectively); the bla-ROB-1 was identified in 29% of *M. haemolytica* isolates. Similar findings have been reported from other respiratory isolates of cattle. One study reported that 0 of 48 respiratory isolates from cattle demonstrated phenotypic AMR to ceftiofur based on MIC testing; however, the prevalence of the genes bla-OXA-2 and bla-ROB-1 were approximately 100% and 82%, respectively [36]. Another study of respiratory bacterial isolates from cattle reported 0 of 64 isolates classified as resistant to ceftiofur based on MIC testing, despite finding the bla-ROB-1 gene in study isolates [37]. This marked difference, which appears to be present in multiple studies of bovine respiratory bacterial isolates, may be due to AMR genes not being expressed, AMR genes improperly predicted to confer resistance to cephalosporins, conference of resistance being limited to older generations of cephalosporins, or due to laboratory testing conditions that do not reflect biologic conditions in which resistance may occur. As an example of the latter, if the breakpoint used for determining resistance from susceptibility testing is much higher than the concentration that separates wild-type isolates from those with acquired genetic elements that confer AMR (such as when using an epidemiologic cutoff), a phenotype of resistance based on the clinical breakpoint may not be determined by this test. The clinical breakpoint values for ceftiofur for BRD pathogens were set in 1988, prior to the establishment of the CLSI veterinary antimicrobial susceptibility testing subcommittee, and used peak total serum drug concentrations (rather than unbound drug) following systemic administration, along with MIC90 values against BRD pathogens to set susceptible, intermediate, and resistant breakpoints (personal communication, Mike Sweeney and Jeff Watts, 11/7/23). This legacy clinical breakpoint was based solely on pharmacokinetic data. Current standards now integrate the microbiologic, pharmacokinetic, and pharmacodynamic data based on the approved dosing regimens and clinical outcome data, to establish a clinically relevant and host-disease-specific clinical breakpoint [66,67,68]. An epidemiologic breakpoint for ceftiofur in bovine respiratory bacterial isolates is not available. A 2016 study investigated the label dose of ceftiofur crystalline-free acid (long-acting formulation of the drug) in plasma, interstitial fluid (ISF), and pulmonary epithelial lining fluid (PELF) [69]. The mean maximum concentration reported in plasma was 4.26 μg/mL; however, the mean maximum concentrations in ISF and PELF reported were 0.2 and 2.09 μg/mL, respectively. An early study of ceftiofur sodium in 1996 investigated MICs for *P. multocida*, *M. haemolytica*, and *H. somni* isolates from cattle; the study reports a mode MIC90 of <0.0039, 0.0015, and <0.0019 μg/mL, respectively [70]. Using the data reported in the 2016 study, ISF concentrations do not reach the breakpoint used to define susceptibility of 2 μg/mL, and only in some animals does the PELF reach concentrations above 2 μg/mL [69]. Historic MIC90 data demonstrate relatively low concentrations effective in inhibiting bacterial growth [70]. It is possible that the CLSI breakpoint of 2 μg/mL may be so high that even with some acquired resistance mechanisms, the Pasteurellaceae investigated may not survive under in vitro testing of relatively high drug concentrations. When treating a disease or applying selective pressure for AMR in vivo, drug concentrations in the tissue or fluid compartment at the site where bacteria are located are likely more representative of the local environment for the selection of AMR than serum values. Ceftiofur concentrations are lower in PELF, ISF, and bronchial secretions than plasma [69,71]. Thus, the AMR genes identified in this study and others may provide an advantage in vivo with selective pressure from the use of AMD that reaches lower concentrations at the site of bacterial growth than the in vitro breakpoints reflect. This mismatch between breakpoint testing and in vivo drug concentrations at the site of interest is one possible explanation for the discordance identified between phenotypic and genotypic AMR for ceftiofur in this study, and other studies of bovine respiratory bacteria.

Discordance was also identified when MDR was compared between phenotype and genotype. The MDR potential of isolates was underestimated by its phenotype compared to the genotype. The process of WGS demonstrates all known AMR genes present in the isolate, even if they are not currently being expressed. In a similar study on Salmonella from small ruminants in Peru, partial concordance between the genotype and phenotype was identified. However, investigators also found some AMR was not predicted by the genotype [31]. A global study of AMR in Salmonella typhoid also found a similar result [28], suggesting that phenotype determined by MIC is not only conferred by known genes and may include yet-to-be-discovered regulatory methods.

Various factors [72,73] are important for the expression of AMR genes, which may not be accurately reflected in culture and susceptibility testing conditions. Susceptibility testing may determine the in vitro susceptibility of the isolate at the time of sampling, but does not demonstrate the full AMR potential of the isolate. The clinical implication of these differences between testing modalities depends on the clinical question being investigated by testing. If clinicians or investigators are more interested in the AMR potential of an isolate or sample, WGS will likely yield more information, with the possible current exception of fluoroquinolones. If clinicians are more interested in the active expression of AMR in vitro, susceptibility, and MIC breakpoint interpretation testing may be more useful. Methods to determine AMR gene expression in samples using RNA are more recently available, however, due to some sample handling requirements, are not widely used yet.

Statistical models for assessing the likelihood that correlations between genotype and phenotype occurred by chance, have limitations. Comparisons with a perfect positive correlation (MCC = 1) between genotype and phenotype for a specific combination of AMR gene and AMR phenotype could not be assessed for significance using McNemar’s test due to lack of discordant data (sum of genotype positive phenotype negative and genotype negative phenotype positive is zero), rendering the test statistic inestimable. Alternative tests of homogeneity were not appropriate for use due to the violation of their assumption of independence, which may result in biased interpretations. Exploring novel methods to compare genotypic and phenotypic AMR traits on a continuous scale rather than as the presence or absence dichotomy may reduce inestimable test statistics and better reflect the biological complexity of drug resistance in bacterial pathogens.

The use of MALDI-TOF for bacterial ID has previously shown limited concordance with WGS in Campylobacter isolates [40]; the present study identified a small number of isolates with evidence of contamination from other respiratory isolates on WGS that were identified by MALDI-TOF as pure cultures. Although this was infrequent, clinicians and researchers should be aware of occasional contamination from morphologically similar bacterial isolates that may not always be detected by MALDI-TOF.

The DNPS samples upper airway bacteria and, although it has been demonstrated to correlate with bacteria found in the lower airways, it is not a direct test of lower airway bacteria identification. This study selectively isolated *P. multocida*, *M. haemolytica*, and *H. somni* from the upper airway; these three Pasteurellaceae are only a fraction of the bacteria that make up the upper respiratory microbiome and thus do not necessarily represent all species or resistance genes present in this biologic niche.

Prevalence data should be interpreted in light of sample size, which was broken down by species. However, with nearly 100 isolates for each species, this appears to be an adequate survey of the target organisms from the sample site when compared with other studies using WGS in bovine respiratory bacteria [36,37]. Quality control allows for population-level analyses, so it is also possible that some individual isolate genomes may have a small probability of a gene going undetected by WGS. The use of MIC testing as the in vitro assay used to determine phenotypic AMR also has limitations, including limited test ranges and breakpoints that may or may not match up well with the environment where bacteria live in the host, and the lack of breakpoints for some AMD. Additionally, there are many factors that influence the expression of AMR genes in bacterial isolates, and specific testing and control of all factors known to affect gene expression was outside the scope of this study.

The high prevalence and frequent co-occurrence of AMR genes in this population suggest that the genetic potential for AMR is high; the previous MIC testing results demonstrate that AMR phenotypes are common. Frequent co-occurrence of AMR genes suggests that efforts to simply change drug use from one class to another class may not decrease AMR as intended. Thus, efforts to prevent BRD, and to decrease reliance on AMD may be particularly valuable in this population of animals. Technologic advances that increase the accessibility of genomic methods of AMR identification and improvements in phenotypic tests of AMD susceptibility may help inform AMR research and judicious AMD use.

## 5. Conclusions

This study aimed to determine the prevalence of AMR genes in Pasteurellaceae sampled from the upper airway of weaned dairy heifers and to determine the concordance between AMR genotype and phenotype in study isolates. Genes that predict the conference of AMR to multiple medically important AMD, which are also used in calf rearing, were highly prevalent in the study population. Concordance between phenotype and genotype of AMR varied depending on the AMD and species tested. Concordance between genotype and phenotype was particularly poor for fluoroquinolone class AMD (enrofloxacin and danofloxacin) and the drug ceftiofur. The lack of excellent concordance suggests these tests cannot be interpreted interchangeably for all AMD in these three respiratory Pasteurellaceae. It is unlikely that either method (WGS or susceptibility testing) of testing for AMR in select isolates represents a perfect prediction of AMR in vivo.

## Figures and Tables

**Table 1:** Matthew’s correlation coefficient values, sensitivity, and specificity of identifying respiratory bacterial isolate resistance by AMR gene identification using WGS compared to CLSI breakpoint interpretation in macrolide class AMD. Where discordant cell counts were <24, McNemar’s exact binomial test was used, demarcated by *. Data from *H. somni* were excluded from the table due to MCC values consistently below 0.25. NDP = no discordant pair; both discordant cells (false positives and false negatives) have zero counts, rendering McNemar’s test statistic inestimable.

Drug	Isolate Species	AMR Genes	Matthew’s Correlation Coefficient	Sensitivity	Specificity	McNemar’s *p* Value
Tilmicosin	*P. multocida*	*erm42*	0.96	0.98	1	0.5 *
		*mphE*	0.15	0.09	1	<0.001
		*msrE*	0.15	0.09	1	<0.001
	*M. haemolytica*	*erm42*	0.40	0.36	1	<0.001
		*mphE*	0.20	0.11	1	<0.001
		*msrE*	0.20	0.11	1	<0.001
Gamithromycin	*P. multocida*	*erm42*	0.92	1	0.89	0.125 *
		*mphE*	0.17	0.10	1	<0.001
		*msrE*	0.17	0.10	1	<0.001
	*M. haemolytica*	*erm42*	0.70	0.61	1	<0.001 *
		*mphE*	0.36	0.20	1	<0.001
		*msrE*	0.36	0.20	1	<0.001
Tulathromycin	*P. multocida*	*erm42*	0.26	1	0.31	<0.001
		*mphE*	0.60	0.41	1	<0.001 *
		*msrE*	0.60	0.41	1	<0.001 *
	*M. haemolytica*	*erm42*	0.83	0.76	1	0.008 *
		*mphE*	0.42	0.24	1	<0.001
		*msrE*	0.42	0.24	1	<0.001
Tildipirosin	*P. multocida*	*erm42*	1	1	1	NDP
		*mphE*	0.16	0.09	1	<0.001
		*msrE*	0.16	0.09	1	<0.001
	*M. haemolytica*	*erm42*	0.50	0.42	1	<0.001
		*mphE*	0.26	0.14	1	<0.001
		*msrE*	0.26	0.14	1	<0.001

## Data Availability

Genomic and antimicrobial susceptibility data used in the data analysis of this manuscript have been made publicly available, including genome sequences uploaded to the Sequence Read Archive, with no confidential metadata included in the study analysis or the public data files. This study was funded by the Antimicrobial Use and Stewardship (AUS) Program of the California Department of Food and Agriculture (CDFA) and is subject to California Food and Agricultural Code (FAC) Sections 14400 to 14408. FAC Section 14407 requires that data collected be held confidential to prevent the individual identification of a farm or business.

## Supplementary Materials

The following supporting information can be downloaded at: https://www.mdpi.com/article/10.3390/pathogens13040300/s1, Material S1: Methods for Culture and Susceptibility Testing; Table S1: Standard Drug Classes Used for AMR Gene Classification; Table S2: Summary Statistics for AMR Gene ID vs. Culture and Susceptibility Testing for Identification of AMR; Table S3: QC Metrics; Table S4: MALDI vs. culture ID. MALDI-TOF bacterial isolate identification (ID) compared to genomic ID from WGS.
