# Supplementary material for: Antimicrobial Resistance Genes in Respiratory Bacteria from Weaned Dairy Heifers
Source: Pathogens. 2024 Apr 3;13(4):300. doi: 10.3390/pathogens13040300 (PMC11053459; doi:10.3390/pathogens13040300)
Supplement: Supplementary file 1 [file pathogens-13-00300-s001.zip › Material S1 information_ culture and susceptibility methods.pdf]

Supplementary Material

S1: Methods for Culture and Susceptibility Testing

Culture and antimicrobial sensitivity testing was performed as previously described [3]. The following description is slightly modified from the previously published text to most clearly describe the culture and sensitivity testing performed on samples used in this study; extraneous information regarding additional samples that pertained only to the previous study was removed. The previous publication [3] should be consulted for original citations.

All swabs were submitted to the California Animal Health and Food Safety laboratory in Davis, CA for selective culture and sensitivity testing. Each DNPS in Amies with charcoal transport media was cultured on sheep blood-3% agar (3% SBA) and chocolate agar (CHOC). Plates were incubated for 48 hours at 35 ± 2˚C in 5-10% CO_2 (3% SBA, CHOC) and examined every 18-24 hours for colonies of interest. Organisms of interest included the respiratory pathogens *Mannheimia haemolytica*, *Pasteurella multocida*, and *Histophilus somni* recovered from DNPS. All colonies of interest were confirmed by biochemical testing and matrix-assisted, laser desorption-ionization time of flight (MALDI-TOF) mass spectrometry.

Antimicrobial susceptibility testing was performed using broth microdilution (Trek Sensititre, Trek Diagnostic Systems, Thermo Fisher Scientific, Waltham, MA) according to Clinical Laboratory Standards Institute (CLSI) guidelines to determine the MIC of the 19 AMD contained on the Sensititre Bovine BOPO7F Vet AST plate (Thermo Scientific, Remel Inc., Lenexa, KS, USA). This panel of 19 AMD was selected to match those monitored by the United States Department of Agriculture, Animal and Plant Health Inspection Service. Where available, interpretive criteria from CLSI-established clinical breakpoints were used to classify an organism as susceptible versus not susceptible (resistant or intermediate). Although there are published CLSI breakpoints for ampicillin and sulfadimethoxine for *P. multocida*, *M. haemolytica*, and *H. somni*, the current MIC breakpoints are below the lowest concentration of drug tested in the standard broth microdilution method used, and thus susceptibility could not be meaningfully interpreted based on the MIC provided. Isolates were tested against antimicrobials at the following dilutions: ampicillin (0.25-16µg/mL), penicillin (0.12-8 µg/mL), ceftiofur (0.25-8 µg/mL), florfenicol (0.25-8 µg/mL), tylosin (0.5-32 µg/mL), tilmicosin (2-16 µg/mL), tulathromycin (8-64 µg/mL), tildipirosin (2-16 µg/mL), gamithromycin (1-8 µg/mL), tiamulin (0.5-32 µg/mL), clindamycin (0.25-16 µg/mL), danofloxacin (0.12-1 µg/mL), enrofloxacin (0.12-2 µg/mL), trimethoprim-sulfamethoxazole (2/38 µg/mL), sulfadimethoxine (256 µg/mL), tetracycline (0.5-8 µg/mL), gentamicin (1-16 µg/mL), neomycin (8-32 µg/mL), and spectinomycin (8-64 µg/mL). *Escherichia coli* ATTC 25922, *Pseudomonas aeruginosa* ATTC 27853, *Enterococcus faecalis* ATTC 29212, and *Staphylococcus aureus* ATTC 29213 were used as quality control organisms.

Isolates were sub-cultured on SBA (*M. haemolytica, P. multocida.)* or CHOC (*H. somni)* and incubated for 18-24 hours at 35˚C in 5-10% CO_2. Each isolate was suspended in 0.85% saline to a concentration equivalent to a 0.5 McFarland standard and added to cation-adjusted Mueller-Hinton broth containing lysed horse blood (*M. haemolytica*, *P. multocida)*, or veterinary

fastidious media (*H. somni)* to achieve 5 X 10^5-1 × 10^6 cfu/mL. Susceptibility plates were incubated for 18-24 hours at 35 ± 2˚C in ambient air (*M. haemolytica*, *P. multocida),* or 5-10% CO_2 (*H. somni)* and observed for visible growth. The MIC was determined as the lowest concentration of antimicrobial that prevented growth.

Multi-drug resistance was defined as a respiratory isolate and AMD test combination for which there were applicable CLSI breakpoints and the isolate was not susceptible to ≥ 3 AMD classes, as previously described. The 11 AMD with applicable CLSI breakpoints represented 7 classes of AMD. Class-wide susceptibility applied when isolates were susceptible to all AMD tested within a class; lack of class wide susceptibility was applied when an isolate was not susceptible (resistant or intermediate) to one or more of any of the drugs tested within the class.

References:

3. Depenbrock S, Aly S, Wenz J, Williams D, ElAshmawy W, Clothier K, et al. In-vitro antibiotic resistance phenotypes of respiratory and enteric bacterial isolates from weaned dairy heifers in California. PLOS ONE. 2021 Nov 24;16(11):e0260292.
