# Supplementary material for: Antimicrobial Resistance Genes in Respiratory Bacteria from Weaned Dairy Heifers
Source: Pathogens. 2024 Apr 3;13(4):300. doi: 10.3390/pathogens13040300 (PMC11053459; doi:10.3390/pathogens13040300)
Supplement: Supplementary file 1 [file pathogens-13-00300-s001.zip › Table S1_drug classes.pdf]

Supplementary 2
Table: Standard drug classes used for AMR gene classification

Standardized Drug Class Categories	Sub-Classes	AMR Genes Present	Drugs tested with clinically applicable breakpoints	Drugs tested without clinically applicable breakpoints
Aminoglycoside		AAC(3)-IV, aadA25, ANT(2'')-Ia, APH(3')-Ia, APH(3'')-Ib, APH(4)-Ia, APH(6)-Id	Gentamicin, Spectinomycin	Neomycin
Beta-Lactam	Cephalosporin(**C**), Penicillin(**P**)	PBP3_mut, OXA-2, OXA-192, ROB-1, ROB-2, ROB-5, ROB-7	Ceftiofur(**C**), Penicillin(**P**)	Ampicillin(**P**)
Macrolide+	Macrolide(**M**), Lincosamide(**L**), Streptogramin	Erm(42), ErmF, mphE(**Macrolide only**), msrE	Gamithromycin(**M**), Tildipirosin(**M**), Tilmicosin(**M**), Tulathromycin(**M**)	Clindamycin(**L**), Tylosin(**M**)
Phenicol		CAT, floR, pp-flo	Florfenicol	
Sulfonamide		sul2		Sulphadimethoxine, Trimethoprin-sulfameth
Tetracycline		tet(D), tet(H)	Tetracycline	
Fluoroquinolone			Danofloxacin, Enrofloxacin	
Pleuromutilin				Tiamulin
Diaminopyrimidine		dfrA14		
Elfamycin		EF-Tu_mut		
