# Supplementary material for: Antimicrobial Resistance Genes in Respiratory Bacteria from Weaned Dairy Heifers
Source: Pathogens. 2024 Apr 3;13(4):300. doi: 10.3390/pathogens13040300 (PMC11053459; doi:10.3390/pathogens13040300)
Supplement: Supplementary file 1 [file pathogens-13-00300-s001.zip › Table S4_ MALDI vs Culture ID.pdf]

Supplementary 5

MALDI-TOF bacterial isolate identification (ID) compared to genomic ID from WGS. Six out of 130 *P. multocida* cultures were identified as pure *P. multocida* by MALDI-TOF, however genomic ID using WGS demonstrated evidence of contamination. The isolate ID provided by MALDI-TOF is highlighted in blue.

**Organism**	1	2	3	4	5	6
**CheckM Predicted %Contamination**	101	100	84	58	44	18
P. multocida	79	90	94	95	95	2
M. haemolytica	20	0	5	0	5	0
H. somni	0	0	0	0	0	97
Moraxella bovoculi	0	10	0	5	0	0
